# Exploring Patients’ Experiences of Living With Liver Cirrhosis-Related Ascites and Receiving Follow-Up Care in Nurse-Led Outpatient Clinics

**DOI:** 10.1177/23333936261444731

**Published:** 2026-04-26

**Authors:** Sigurd Myhre Halvorsen, Marit Hegg Reime

**Affiliations:** 1 Lovisenberg Diaconal Hospital, Oslo, Norway; 2 Western Norway University of Applied Sciences, Bergen, Norway; 3 Lovisenberg Diaconal University College, Oslo, Norway

**Keywords:** ascites, chronic illness, dignity, embodied knowledge, liver cirrhosis, patient experience, person-centred care, reflexive thematic analysis, qualitative research, Norway

## Abstract

In Europe the incidence of patients living with liver cirrhosis is growing due to increasing alcohol consumption, increased life span and higher body mass index. Patients with decompensated liver cirrhosis are a vulnerable group of patients suffering from stigma, negative stereotyping and bodily limitations, needing close follow-up care through a holistic nursing care model. This study aimed to explore patients’ experiences of living with cirrhosis-related ascites, and the follow-up care they receive in nurse-led outpatient clinics. A qualitative approach was employed using semi-structured individual interviews and inductive reflexive thematic analysis. Eight interviews were conducted and our analysis generated two major themes: “Coming to terms with the impact of ascites” and “My second home–building partnership roles.” The first theme comprised three sub-themes related to dignity, embodied knowledge and loss of energy. The second theme comprised two sub-themes related to continuity of care and patient involvement in their care. The study provides valuable insights on how nurse-led follow-up care, through continuity, availability, commitment, patient education and patient involvement may relieve the anxiety and distress this patient group may experience due to having a serious disease with an unpredictable trajectory.

## Introduction

The incidence of liver cirrhosis is rising in Europe due to increasing alcohol consumption, longer lifespans, and higher body mass index (Hamberg et al., 2023). The main causes of cirrhosis are excessive alcohol use (45%), hepatitis C (41%), and metabolic dysfunction-associated steatotic liver disease (MASLD, 26%) (European Association for the Study of the Liver (EASL), 2024; Tapper & Parikh, 2023). Annually, around 2 million deaths worldwide are linked to liver disease: 1 million from cirrhosis and 1 million from viral hepatitis and hepatocellular carcinoma, and over 60% of these deaths occur in men (Sepanlou et al., 2020). Cirrhosis ranks as the 11th most common cause of death and the third leading cause for those aged 45 to 64, accounting for 3.5% of all deaths globally (Kim et al., 2021). The global burden of advanced MASLD is set to more than double from 2016 to 2030 due to rising metabolic risk factors and aging populations (Estes et al., 2018). In Norway, predictions indicate that 1% to 1.5% of Norwegians will experience liver failure in their lifetime (Norwegian Health Informatics, 2023), with a similar increasing trend in Sweden and other high-income countries in Europe (Vaz et al., 2025).

Liver cirrhosis involves the fibrotic replacement of normal liver tissue due to chronic liver disease (Tapper & Parikh, 2023). It is often asymptomatic until complications arise, known as decompensation, leading to symptoms like recurring ascites, variceal bleeding, and hepatic encephalopathy (Hamberg et al., 2023; Jepsen et al., 2010). Ascites, the most common complication, significantly impacts patients’ quality of life (Hamberg et al., 2023; Rudler et al., 2020; Tapper & Parikh, 2023) and can cause abdominal discomfort, dyspnea, umbilical changes, hernias, and scrotal edema (Krige & Beckingham, 2001). Stiffness and dullness may occur with fluid accumulation of around 1.5 liters or more, particularly in obese individuals (Aithal et al., 2021).

Ascites, which has various causes including cirrhosis (55%), cancer (29%), cardiac failure (6%), end-stage renal disease (3%), and other factors (7%) (Aithal et al., 2021; Rudler et al., 2020), is categorized into three grades based on severity: (1) Grade 1 (Mild) is detected only by ultrasonography and requires a low-sodium diet for management, (2) Grade 2 (Moderate) is characterized by abdominal distension and discomfort, this grade necessitates sodium restriction and the use of diuretics to manage symptoms, and (3) Grade 3 (Severe) is marked by tense abdominal distension and a fluid wave, requiring paracentesis in a hospital setting for treatment (Angeli et al., 2018; Piano et al., 2018). If more than 5 liters of fluid are removed, patients receive intravenous protein solutions to prevent dehydration (Aithal et al., 2021). In addition to these specific treatments, fluid restriction is recommended only in cases of moderate to severe hyponatremia, defined as a serum sodium level of ≤ 125 mmol/L (Biggins et al., 2021). If these symptomatic treatments are ineffective, more invasive options such as transjugular intrahepatic portosystemic shunt (TIPS) or liver transplantation may be necessary (Tapper & Parikh, 2023).

Patient experiences are vital in nursing care and should guide the development of follow-up services (Arman et al., 2015; McAfee, 2012). To our knowledge, the experiences of patients with ascites from liver cirrhosis and the follow-up care in nurse-led outpatient clinics need further exploration.

### Background

Patients with decompensated liver cirrhosis constitute a particularly vulnerable group, often experiencing social exposure and stigma from others. This reality creates a critical demand for close follow-up and support from the healthcare system (Hamberg et al., 2023). Research indicates that these patients frequently encounter stigma manifested through labeling, negative stereotyping, hierarchical perceptions, physical limitations, and social loneliness (Hjorth et al., 2023). In light of these challenges, outpatient nursing care has proven beneficial, enhancing self-care and disease management skills, reducing hospital admission rates, improving mortality rates, and ultimately fostering better quality of life, self-efficacy, and patient knowledge (McAfee, 2012; O’Connell et al., 2023). A key factor in promoting improved self-care is the development of patients’ embodied knowledge, which encompasses their personal understanding and awareness of their bodies, coping mechanisms, and overall health (Heggdal, 2013). This concept is crucial for empowering patients to navigate the complexities of their condition effectively. The theory of body-knowledging is particularly relevant to this study, as cultivating embodied knowledge allows patients to articulate their experiences, symptoms, and needs more clearly, leading to more personalized care (Heggdal, 2013). Further supporting this need for personalized care, Ramachandran et al. (2022) found that patients highly valued nurse-led clinics for their accessibility, tailored information, and the nurse-patient relationship that embodies person-centred care (PCC). PCC is especially significant for patients with liver cirrhosis and ascites, given the frequent fluctuations in their health status (Hjorth et al., 2020). It has been recognized as a vital component of healthcare quality and patient safety (Santana et al., 2018), encompassing empathy, respect, engagement, effective communication, shared decision-making, and a holistic focus on coordinated care (Eklund et al., 2019; Morgan & Yoder, 2012).

The philosophical framework of nursing articulated by the Norwegian philosopher Kari Martinsen (2006) further deepens our understanding of care. She identifies three dimensions of caring: relational, practical, and moral, with the moral dimension being paramount due to the inherent power dynamics between caregivers and patients. Central to Martinsen’s philosophy is the concept of solidarity with vulnerable patients, which aims to establish a morally responsible power relationship. In outpatient clinics, cooperation and genuine engagement are essential for fostering a supportive environment, thereby laying the groundwork for patients’ learning and coping processes (Alvsvåg, 2018; Heggdal, 2013).

Literature search in Cinahl, MEDLINE, Embase, and PsycInfo revealed only two studies exploring patients’ experiences living with ascites, one exploring patient experiences with benign ascites Day et al. (2015) and one with malign ascites (Day et al., 2013). Day et al. (2015) highlight that discomfort and limitations from non-malignant ascites adversely affect daily functioning and well-being. Similarly, Day et al. (2013) emphasize that these physical manifestations can lead to a profound sense of loss of control over one’s body and life. Qualitative studies on patient experiences living with liver cirrhosis face a multifaceted and often challenging experience that significantly impacts their physical, emotional, and social well-being. Abdi et al. (2015) highlight that individuals often feel a profound sense of vulnerability due to the unpredictable nature of the disease, which is echoed in the findings of Hjorth et al. (2020) who describe life with cirrhosis as akin to an “unpredictable roller coaster.” This unpredictability contributes to anxiety, as patients anticipate potential exacerbations and complications associated with their condition.

Patients frequently experience stigma and social isolation stemming from their diagnosis. Fagerström and Frisman (2017) articulate that this stigma can lead to feelings of shame and withdrawal from social interactions, further exacerbating their emotional distress. The study by Namaghi et al. (2022) reinforces this notion by showing that societal perceptions can diminish the quality of life, creating barriers to support and understanding from family and friends.

Patients’ experiences with healthcare services also profoundly influence their journey with cirrhosis. Hjorth et al. (2023) emphasize the importance of feeling safe within the healthcare system, stating that patients often oscillated between feeling well-supported and experiencing care as fragmented or inadequate. This variability in healthcare experiences can lead to frustrations and a sense of vulnerability as patients navigate complex medical environments. The findings from Carbonneau et al. (2018) regarding patient views on advance care planning illustrate the desire for more proactive engagement and communication with healthcare providers to better manage future health decisions. Kimbell and Murray (2015) underscore that patients prioritize person-centred care that addresses not only medical needs but also emotional, social, and spiritual aspects. Understanding each patient’s unique situation facilitates a more supportive framework for managing their condition effectively.

In summary, patients living with cirrhosis navigate a complex landscape characterized by emotional distress, social stigma, physical burdens, and varying experiences with healthcare.

To obtain a deeper understanding on how health services can improve and adjust their follow-up care for this patient group a user perspective including patients’ preferences is crucial (Hamberg et al., 2023). In light of the increasing number of patients suffering from cirrhosis and ascites, it is essential to understand their experiences of illness beyond the boundaries of medical care, thus ensuring that their holistic needs are adequately addressed (Kimbell & Murray, 2015). Given the lack of research on the perspectives of cirrhotic patients’ experience living with ascites (Namaghi et al., 2022; Ramachandran et al., 2022), this study may contribute to inform improvements in outpatient clinical practice for this complex patient group. Therefore, this study aims to explore patients experiences of living with liver cirrhosis-related ascites and with the nurse-led outpatient follow-up care they receive.

## Methodology

### Design

A qualitative descriptive design was employed, as this approach is particularly appropriate for generating practice relevant knowledge derived from participants accounts (Bradshaw et al., 2017; Caelli et al., 2003). In the present study, this design facilitated a comprehensive exploration of patients’ experiences of living with ascites related to liver cirrhosis, with attention to the impact on daily life, coping processes, and interactions with healthcare services. Data were collected through semi-structured individual interviews and analyzed using inductive reflexive thematic analysis, enabling identification of patterns of meaning while maintaining analytic rigor and reflexivity (Braun & Clark, 2022).

### Study Setting

This research recruited patients from nurse-led outpatient clinics located in a university hospital and a local hospital in the South-Eastern part of Norway. Each patient received a consultation lasting between 30 and 60 min, 1 h for regular consultations, while shorter appointments were scheduled for medication adjustments. Nurses were responsible for providing follow-up care and adjusting medications in accordance with guidelines set by a hepatologist. The consultation may include an ultrasound to identify ascites, a FibroScan to assess the extent of liver cirrhosis, provide health-related education and counseling to assist patients in avoiding disease triggers such as alcohol, and an evaluation of necessary adjustments to their medication regimen to prevent decompensation. Additionally, the need for paracentesis may also be assessed. Nurses in these outpatient clinics typically hold either a postgraduate education in gastroenterological nursing or a master’s degree, which ensures a high standard of care for this patient population.

In Norway, the healthcare system is funded through taxation, which allows for universal access to services. Patients do not incur direct costs for inpatient care in hospitals, as these expenses are covered by the national health insurance scheme. For outpatient treatment, patients are required to pay co-payments, but there are annual limits on out-of-pocket expenses. Once patients reach this limit, they are exempt from further charges for the remainder of the year. This structure not only ensures accessibility to healthcare services but also promotes responsible resource use.

### Recruitment, Inclusion, and Exclusion Criteria

Nurses working in the outpatient clinics gave patients who fulfilled the inclusion criteria written information about the study and provided contact information to the researcher if patients wanted to participate. Thereafter the primary author contacted these patients by phone to make appointments for the interviews. A purposeful sampling strategy was employed to obtain information-rich cases that included patients varying in age, sex, levels of ascites, etiologies and duration since diagnosis. This approach aimed to ensure a comprehensive representation of the patient group and their experiences during the recruitment process (Polit & Beck, 2021). The included participants were adult patients suffering from decompensated liver cirrhosis with ascites and who received treatment and follow-up care in nurse-led outpatient clinics. Exclusion criteria were patients with compensated liver disease, patients treated for acute liver failure or acute on chronic liver failure, patients who did not speak and understand the Norwegian language or patients who were cognitively impaired.

### Data Collection

After signing the written consent, individual interviews were performed by the primary researcher between October 2024 and February 2025, guided by an interview form (Kallio et al., 2016). The interview guide was based on earlier research and by nurses experiences from consultations with this patient group. In addition, to add further relevance to the research, a pilot interview was performed with a user representative in one of the hospitals, resulting in changing the order of some questions and rephrasing some others. The pilot interview was not included in the study. The primary researcher had no previous care-provider relationships with the participants. Semi-structured interviews were chosen because of their flexible nature guided by the participants responses, and open-ended questions may encourage participants to speak freely with the aim to gain rich data on the participants experiences (Braun & Clark, 2022; Kallio et al., 2016).

Based on each patients wish, two interviews took place in the hospital, five in the privacy of participants home, and one over the phone. The interviews lasted between 22 and 73 min, with a mean of 41 min. The interviews started with repeating information from the consent form about the aim of the study and participants rights. The interview form included topics such as participants’ experiences living with liver cirrhosis-related ascites, challenges associated with this condition, perceptions of follow-up care from nurses in the outpatient clinic, experiences adhering to recommendations, and participants’ wishes for their follow-up care (Appendix 1). During the interview the researcher used non-verbal communication as keeping eye contact and nodding as confirmation of interest, in addition to posing relevant follow-up questions to the participants stories. The interviews were audio recorded, thereafter stored at a secure research server, and immediately transcribed using an artificial intelligence program (University of Oslo, 2025), and anonymized. The researcher controlled the transcribed text to check accuracy and thereafter deleted the recordings from the recorder. The material constituted 342 min audio recordings and 111 single-spaced text pages in Microsoft Word.

### Analysis

An inductive reflexive thematic analysis was employed to identify patterns and themes within the dataset, offering valuable insights into participants’ experiences and meanings. This analysis followed the six steps outlined by Braun et al. (2019), which provide a non-positivist or “Big Q” alternative grounded in constructivist and interpretative qualitative research values. This approach acknowledges researchers’ subjectivity as a valuable resource and emphasizes that knowledge is situated (Braun & Clarke, 2024).

Initially, the first author familiarized themselves with the material by listening to the recorded interviews to gain contextual understanding (Braun & Clark, 2022). Both authors then read the transcribed text multiple times, taking notes to identify patterns and insights. An open coding approach was employed to interpret participants’ communications (Braun & Clark, 2022). In the second phase, initial codes—both semantic and latent—relevant to the study’s aim and theme development were generated. Coding was conducted using the comments function in Microsoft Word, where codes were noted in the margins and relevant text was highlighted. In phase three, all codes were transferred to a spreadsheet for review and analysis to identify how different codes could combine into tentative themes or sub-themes. In the fourth phase, potential themes were reviewed, and in the fifth phase, themes were defined and named by revisiting the transcribed text to ensure coherence with the entire dataset. During these phases, researchers also selected data extracts for the results write-up. Following Braun and Clark (2022), theme development involved creating meaningful names that captured each theme’s central idea (Byrne, 2022). The sixth and final phase involved producing a report of the findings (Braun et al., 2019).

### Ethical Considerations

The study was assessed by the regional ethics committee (ref.739031) deeming it exempts for further approval. The Norwegian Agency for Shared Services in Education and Research (ref.482259) approved the study, as the privacy in research was ensured. In addition, study approval was collected from the participating hospitals through their managers and data protection officers. We complied with the principles of the Helsinki Declaration (The World Medical Association, 2024), and all participants were provided with a form informing them about the aim of the study, and that participation was voluntary without affecting their future care and treatment. Verbal and written consent for participation and for audio recordings of interviews were collected from all participants. During the interviews, communication strategies were employed to mitigate the asymmetrical power relationship between the researcher and the patient (Brinkmann, 2007).

### Rigor and Reflexivity

Reflective thematic analysis recognizes the researcher’s subjectivity and active role in the process of producing knowledge (Braun et al., 2019). Researchers interpret research data based on their cultural history, social positioning, education, experience and theoretical and ideological attitude (Braun et al., 2019). Reflexivity refers to the process of critical self-reflection about oneself as a researcher and one’s influence on the research process (Malterud, 2001). The first author has worked with this patient group for almost two decades in a hospital setting, and a personal interest has arisen from talking to patients, seeing them struggle with both coping and adjusting to their disease. Both authors held a postgraduate education within gastroenterological nursing, but the last author has worked with other patient groups within the gastroenterological field. In our study, credibility was enhanced by recruiting participants who could address the study’s aims and by having two researchers conduct the analysis. By reporting a description of the participants demographics, the setting, the research process, presenting quotations and discussing the findings in relation to other international studies, transparency and transferability was strengthened. The researchers used reflexive journaling to record ideas and personal thoughts and to understand the responses of participants in this study.

## Findings

This study is grounded in semi-structured individual interviews conducted with eight participants, comprising five women and three men, who were living with liver cirrhosis and ascites. The ages of the participants ranged from 36 to 83 years, with a mean age of 67 years. The duration of their illness ranged from 1 to 10 years. Additional characteristics of the participants is shown in Table 1.

**Table 1.** Characteristics of the Participants.

Demographics	Participants
Gender	
Men	3
Women	5
Age	
30–45	1
46–60	1
61–75	1
>75	5
Marital status	
Single	3
Cohabitant	2
Widowed	3
Level of education	
Higher education/university	3
Lower than university level	5
Etiology of cirrhosis	
Alcohol	6
Cancer	2
Time since cirrhosis diagnosis	
<2 years	2
2–5 years	3
>5 years	3
Employment	
Retired	6
Sick leave	1
Disability pension	1

**Figure 1.**
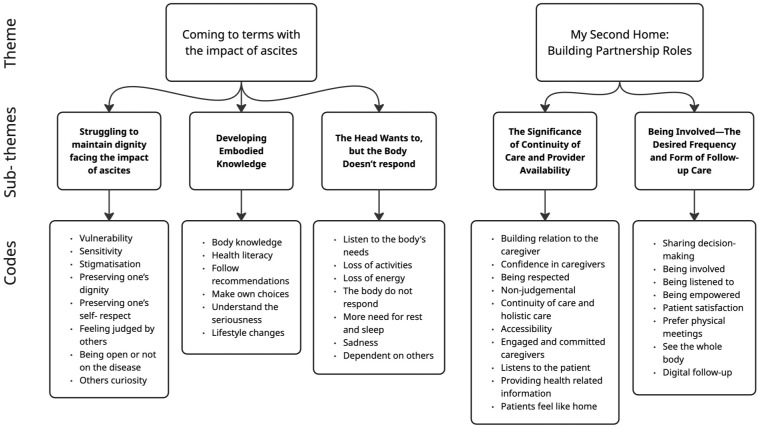
Overview of themes, sub-themes and codes.

The analysis generated two primary themes that reflected the participants’ experiences of living with ascites secondary to liver cirrhosis, as well as their perception of the follow-up care they received in nurse-led outpatient clinics. Figure 1 presents an overview of principal themes, sub-themes, and codes constructed during the analysis.

### Coming to Terms With the Impact of Ascites

The first theme elucidates the participants’ experiences in coming to terms with their illness. It encompasses how they learn to cope—or struggle to cope—with their condition. This theme comprises three sub-themes: “Struggling to maintain Dignity facing the Impact of Ascites,” “Developing Embodied Knowledge,” and “The Head Wants to, but the Body Doesn’t Respond.”

#### Struggling to Maintain Dignity Facing the Impact of Ascites

Participants described ascites as a highly visible and physically burdensome manifestation of liver cirrhosis that profoundly affected their dignity. The markedly distended abdomen, changes in skin and scleral color, and associated limitations in mobility made the illness outwardly apparent to others, often prompting unsolicited attention and socially stigmatizing responses. These visible signs, combined with pain, heaviness, and reduced appetite, created a constellation of challenges that threatened participants’ sense of self-respect in everyday life.

Several participants reported becoming acutely aware of how others reacted to their changed appearance. Being mistaken for pregnant or subjected to staring and whispering generated feelings of embarrassment and vulnerability. One participant explained how such encounters “led to considerable whispering and gossiping,” illustrating how visible bodily changes exposed them to social judgment. Others described situations where immobility due to severe ascites forced them into degrading circumstances, such as being unable to leave the bed during a bowel preparation regimen for a colonoscopy.

I was left with 18 litres of ascites. I was larger than I had ever been. I couldn’t get out of bed. With that abdomen, you simply cannot get up. They suggested things like, “Just do it in the bed.” Do you know how degrading that is? (Participant 2)

Participants also reflected on perceived stigma linked to visible signs of liver disease, particularly yellowing of the eyes, which they believed prompted assumptions about alcohol misuse. As one participant noted, “everyone. . . notices the slight yellow tint and thinks, ‘look how she has ruined her life’” (Participant 2). These perceived judgments intensified feelings of shame and discomfort in social settings. At the same time, some participants described efforts to reclaim dignity through openness about their condition. Being honest about their illness, rather than avoiding conversations about it, was perceived to counter stigma and maintain a sense of control: “I believe in admitting my illness rather than hiding it” (Participant 2). Overall, the struggle to preserve dignity was shaped by both the physical visibility of ascites and the social interpretations attached to it.

#### Developing Embodied Knowledge

Participants described the development of embodied knowledge as a gradual process that began with the initial shock of diagnosis and deepened as they learned to live with the physical realities of ascites. The diagnosis often confronted them with the seriousness of their illness, prompting a new awareness of bodily vulnerability. Honest communication from clinicians—though sometimes unsettling—was experienced as essential in helping participants grasp the implications of their condition. As one participant explained, “I did not fully understand the seriousness until they were brutally honest with me. . . but it was necessary for me to understand my disease” (Participant 2).

As participants navigated treatments and lifestyle modifications, their growing bodily awareness shaped their daily decisions. Guidance from nurses and physicians—on alcohol cessation, sodium and fluid restriction, and medication management—became part of an evolving understanding of how liver disease affected their bodies. For some, adopting abstinence felt like a clear and purposeful commitment, whereas others described tensions between health needs and the social meanings attached to drinking. “When I was diagnosed, I became a total abstainer the very same day. I stopped drinking alcohol entirely–no more compromising my liver! I made a choice, and I don’t miss it at all” (Participant 3). Another participant highlighted the social aspects of drinking: “I have more or less stopped drinking. I occasionally have a light beer; I feel the need to be a little bit social” (Participant 8).

Learning occurred incrementally through repeated clinical encounters, where participants integrated new information with their lived experience. This included becoming attentive to how certain foods, medications, and behaviors affected symptoms. One participant highlighted this ongoing learning process:

There isn’t a single visit where I haven’t learned something new. I didn’t fully understand the purpose of laxatives; I had almost ceased using them. Now I realize their importance in relation to ammonia levels in the body. (Participant 3)

Over time, participants developed practical strategies—monitoring weight and abdominal girth, adapting their diet, and working with nutritionists—reflecting a growing sense of agency in caring for their bodies. This newfound understanding empowered them to make informed dietary choices. For instance, one participant remarked, “I eat caviar because I don’t consume enough fish” (Participant 1), illustrating their proactive approach to supplementing their diet and addressing nutritional needs. Participants shared a myriad of recommendations they received and adhered to, empowering themselves to make autonomous decisions about their health. One participant emphasized:

I have a fluid restriction of 2 liters per day, but I have started drinking a little more. I monitor my waistline and weight. . . I ensure that I consume a minimum of 2000 kcal a day, and I manage well. Together with the clinical nutritionist, I adjust my diet and learn. (Participant 2)

Overall, developing embodied knowledge involved an ongoing negotiation between medical guidance, bodily sensations, and lived experience. Through this process, participants cultivated a deeper understanding of their illness and how to manage it, enabling more informed and autonomous decisions in everyday life.

#### The Head Wants to, But the Body Doesn’t Respond

Participants described a striking mismatch between their intentions and their physical capacity—a form of embodied resistance in which the body no longer aligned with their will. Across interviews, fatigue related to ascites created a pervasive sense of uncertainty, making it difficult to anticipate what they would be able to accomplish on any given day. This unpredictability constrained daily life and forced participants to relinquish activities they once performed routinely. Cycling, swimming, and walking—previously taken for granted—now required conscious calculation or were no longer possible. Increasing problems with balance further reinforced this dependence, prompting several to rely more heavily on others for support.

The data revealed a persistent tension between participants’ desire and their altered bodily capabilities. Despite waking with plans and a desire to engage meaningfully, their bodies often imposed restrictions that redirected or halted those intentions. One participant captured this tension succinctly: “My mind is filled with projects, like organizing my storage room and building shelves. However, the moment I get up, I can feel that my body isn’t cooperating” (Participant 2). This illustrates how participants experienced a profound shift in their bodily functioning, where a once-reliable body became unpredictable and less responsive, demanding continual adjustment in everyday life. To manage this dissonance, participants developed individualized strategies for energy conservation and activity pacing. Many described structuring their days around periods of rest or sleep to maintain enough stamina for essential tasks. This was not merely passive resignation; rather, it represented a deliberate effort to preserve autonomy and maintain continuity with valued aspects of life. For some, long-term planning allowed them to reintroduce hobbies that had been set aside for years. As one participant explained, “Careful preparation enables me to adapt, cope and adjust despite ongoing fatigue” (Participant 3). Yet even with such strategies, participants continued to experience moments of grief when confronted with activities that symbolized their identity or independence. For one participant, maintenance of his boat marked a cherished personal routine. However, the exhaustion that followed even brief attempts to engage in this activity underscored the limitations imposed by his condition; “After starting the task, I had to return home and sleep until the following day before continuing” (Participant 6). This example reflects how fatigue reshaped not only physical capability but also emotional connections to meaningful roles.

For participants of working age, the tension between intention and bodily constraint was especially prominent. One participant had transitioned onto disability benefits, as the fatigue made it impossible to maintain a standard workday. He described feeling “dead tired” (Participant 8), highlighting the totalizing nature of his exhaustion. Another participant expressed a strong desire to return to work in some capacity—not only for structure but to regain a sense of purpose. She hoped that part-time or flexible arrangements could support her engagement in meaningful employment (Participant 2). This narrative reveals how bodily limitations extend beyond physical restrictions to affect identity, social participation, and self-worth.

Overall, this sub-theme captures how ascites-related fatigue generates a fundamental disconnect between the will to act and the body’s diminished capacity. Participants responded with significant effort and strategic adaptation, yet the persistent mismatch between intention and ability contributed to feelings of loss and reorientation of daily life.

### My Second Home–Building Partnership Roles

The second theme examines the relationship between patients and nurses at the outpatient clinic. It delves into participants’ experiences regarding the quality of care during consultations, the availability of providers, and how nurses involve patients in their own care. This theme is organized into two subthemes: “The Significance of Continuity of Care and Provider Availability” and “Being Involved–The Desired Frequency and Form of Follow-up Care.”

#### The Significance of Continuity of Care and Provider Availability

Participants emphasized that continuity of care—particularly through ongoing relationships with the outpatient clinic nurses—was essential in managing the uncertainties associated with ascites. The stability of these relationships fostered trust, reassurance, and a sense of being genuinely cared for. Many participants described the nurses as approachable, attentive, and consistently respectful, qualities that helped counterbalance the unpredictability of their symptoms. Referring to nurses by their first names was common, and this familiarity strengthened a sense of connection. One participant explained that the clinic felt like “A second home, where staff work with their heart and soul, and go the extra mile and see you as a human being. . . not just a number in the statistics” (Participant 3). Such continuity offered more than procedural support—it created a relational environment where patients felt seen and valued.

Before the establishment of nurse-led consultations, participants often struggled with long waiting times for physician appointments, which left them feeling uncertain and unprepared for symptom fluctuations. The introduction of nurse-led hepatic clinics transformed this experience by offering more timely access to care and symptom-specific guidance. Participants valued being able to contact the clinic directly, using the provided phone number to ask questions or request earlier appointments when symptoms worsened. This availability helped restore a sense of control during fluctuating health: “If I feel that something is off, I can simply make a call. It helps to keep me calm” (Participant 4). For many, immediate access to knowledgeable providers was not simply convenient—it was psychologically grounding. Receiving follow-up at the local hospital further enhanced this sense of continuity, particularly because it simplified logistics and reduced the stress of navigating unfamiliar systems. Participants contrasted this experience with encounters in primary care settings, where they sometimes felt overlooked or where interactions felt rushed or impersonal. In contrast, the outpatient nurses devoted time to listening and engaging meaningfully, which deepened patients’ confidence in the care they received. As one participant reflected, “Such relationships built over years are very important” (Participant 1).

Examples of provider dedication reinforced participants’ perception of the nurses’ commitment. One participant described feeling genuinely cared for when a nurse visited her on a different ward while she was hospitalized for a fracture, despite the appointment originally being scheduled at the outpatient clinic. The nurse “Took her time and showed her commitment by keeping the appointment” (Participant 5), which, to the participant, signified a level of care that extended beyond routine responsibilities. Clear, accessible health information also emerged as a critical component of continuity. Participants stressed the importance of receiving guidance in a form they could understand, especially because the complexity of liver disease and ascites could otherwise feel overwhelming. Nurses used visual aids and simple explanations to support patients’ comprehension: “She explains liver illness very well. . . she uses pictures to illustrate what is happening and where” (Participant 7). This educational support allowed patients to feel more confident in recognizing symptoms and managing their condition at home. At the same time, participants differed in how much information they wanted about their prognosis and future possibilities. Some felt they lacked a comprehensive overview and desired more clarity about potential scenarios, particularly as ascites progressed. Others preferred limited information, expressing that too much detail heightened feelings of vulnerability or helplessness, “I’m not really interested in hearing too much. . . you just feel so powerless” (Participant 5).

Overall, the sub-theme illustrates how continuity of care and provider availability served as anchors in a context marked by physical unpredictability and emotional strain. Consistent relationships, timely access, and tailored communication enabled participants to feel supported, informed, and less alone as they navigated the complexities of living with ascites.

#### Being Involved–the Desired Frequency and Form of Follow-Up Care

Participants emphasized that being actively involved in decisions about their follow-up care was central to feeling secure and respected in the management of ascites. Involvement was described as more than having choices offered—it represented an opportunity to influence the structure and content of their care. This sense of partnership enhanced patients’ confidence, contributing to a perception of follow-up as something collaborative rather than prescriptive. One participant described this as a “luxury experience,” reflecting the empowerment she felt when allowed to determine the format and sequencing of her consultations, such as deciding whether a session with the nutritionist should be conducted in person or by phone (Participant 3). Such experiences illustrate how shared decision-making helped reinforce patients’ autonomy at a time when their illness imposed restrictions in many other domains of life.

Participants’ preferred frequency of follow-up varied considerably, reflecting differences in disease duration, ascites severity, and personal coping strategies. For some, more frequent contact offered reassurance and helped them detect changes in fluid retention early. Others felt that extended intervals—such as every 6 months—were sufficient and aligned with their desire for stability rather than ongoing medical attention. As one participant expressed, “Routine follow-up at least biannually, or quarterly if needed, contribute to a sense of being adequately supported while avoiding unnecessary appointments” (Participant 8). These variations underscore the importance of tailoring follow-up frequency to individual needs rather than applying a standardized schedule.

Face-to-face consultations were highly valued, particularly because they enabled direct discussions of symptoms and provided opportunities for physical examination—an essential component of managing ascites. Participants consistently highlighted the reassurance gained from in-person encounters, where nuanced symptom descriptions could be conveyed, and bodily changes could be assessed. However, they also recognized the evolving role of digital communication in healthcare. While screens and virtual tools were viewed as increasingly relevant, participants described them as insufficient substitutes for in-person contact. One participant articulated this sentiment by acknowledging the practicality of digital communication while still emphasizing the irreplaceable value of physical presence: “It is not the same. . . there is something about being confined within four walls” (Participant 1). This reflects a deeper need for relational closeness, embodied interaction, and the sense of being genuinely attended to—needs not fully met by technology alone.

Overall, participants’ preferences regarding involvement, frequency, and mode of follow-up care reveal a desire for personalized, flexible support that aligns with their lived realities. Involvement in decision-making fostered a sense of agency, while the ability to adjust follow-up intervals and choose between in-person and digital contact helped participants feel that their care was responsive rather than routine. These elements combined to create a follow-up experience that recognized them not merely as patients to be monitored, but as partners whose perspectives, comfort, and preferences were integral to effective ascites management.

## Discussion

### Discussion of Findings

This study advances the understanding of living with ascites by offering an interpretive account grounded in patients’ embodied experiences, care relationships, and preferences for involvement in follow-up care. These findings contribute conceptually to the broader literature on chronic illness and dependency by demonstrating how structural aspects of care—such as accessible nurse-led clinics and shared decision-making—can mediate the emotional and practical consequences of an unpredictable condition. Two main themes answer to the study’s aim of describing patients’ experiences living with liver cirrhosis-related ascites and with the nurse-led outpatient follow-up care and will be discussed in the following.

#### Coming to Terms With the Impact of Ascites

Our study illustrates the significant impact of severe ascites not only on physical health but also on the psychosocial well-being of participants, particularly through feelings of stigmatization. Stigma can be understood as a complex social cognitive process. Public stigma refers to the negative attitudes that society may harbor toward individuals suffering from alcohol-related liver disease, while self-stigma involves the internalized shame that patients may feel about their condition (Schomerus et al., 2022). Participants in our study poignantly described a perceived need to combat the injustices associated with the visible manifestations of their disease, particularly the pronounced abdominal distension that can make them look pregnant. This visible change in bodily appearance often led to feelings of vulnerability, even as they endeavored to maintain their self-respect and dignity.

The concept of dignity is central to how individuals relate to themselves and others, encompassing the inherent value and worthiness that they feel as members of society (Staats et al., 2020). The sense of exposure associated with such visible symptoms aligns with findings by Fagerström and Frisman (2017), which indicate that this stigma can impose significant psychological burdens, exacerbating mental and emotional distress, a concern also highlighted by Kimbell and Murray (2015). Participants in our study expressed differing attitudes about how openly one should discuss their illness. For example, one participant conveyed a reluctance to talk about her condition but emphasized that she did not feel ashamed. In contrast, another participant believed that explicitly acknowledging her disease was vital to preserving her dignity. Interestingly, none of our participants expressed shame about their condition, which stands in contrast to the findings of Hjorth et al. (2020) and Fagerström and Frisman (2017), where a majority of respondents reported feelings of shame related to their illness. This divergence suggests that attitudes toward liver disease and its visibility may vary widely among individuals and underscores the necessity for tailored support that addresses both the physical and emotional complexities of living with severe ascites. The psychosocial ramifications of severe ascites reflect a broader societal issue of stigma, highlighting the need for increased understanding and sensitivity in how we address the experiences of this patient population. By fostering an environment that values openness and dignity, we can better support individuals in navigating the challenges of their condition.

Participants in our study frequently described the diagnosis of cirrhosis as a profound shock, a sentiment that reflects the commonly asymptomatic nature of this disease until it reaches the stage of decompensation (Hamberg et al., 2023). This sudden revelation often forces individuals to confront the reality of their condition and the lifestyle changes that may be necessary to manage it effectively. Despite the initial shock, participants demonstrated a strong willingness to educate themselves about their illness and to make significant lifestyle adjustments aimed at alleviating their symptoms. Many reported completely abstaining from alcohol, while others opted to moderate their intake, particularly in social settings. This shift reflects a crucial step toward taking control of their health. Remarkably, as participants navigated their condition, they developed a heightened sense of bodily awareness—an understanding of how their bodies reacted to different foods and the importance of adhering to sodium and fluid restrictions based on their daily condition. This aligns well with the theory of body-knowledging, which emphasizes the use of bodily knowledge to facilitate health-related changes and to recognize the body’s signs and limits of tolerance (Heggdal, 2013). The ability to interpret bodily cues and adjust behaviors accordingly is vital for managing a chronic condition like cirrhosis, where individualized care and awareness can greatly influence health outcomes. Participants’ engagement in this process underlines the importance of empowering individuals with knowledge about their body, enabling them to make informed decisions that enhance their well-being. While the diagnosis of cirrhosis may be a shocking and challenging experience, the proactive approach adopted by participants reflects resilience and a commitment to improving their health through lifestyle changes and increased bodily awareness.

Participants in our study reported experiencing significant fluctuations in energy levels, often accompanied by a pervasive sense of fatigue that frequently necessitated increased rest and daytime sleep. Despite their motivation to engage in various tasks, many found that their bodies did not respond as desired. This disconnect between intention and physical capability led participants to develop coping strategies, such as becoming attuned to their bodies’ signals and planning social activities well in advance to accommodate their fluctuating energy levels.

The profound sense of tiredness experienced by participants echoes findings from other studies, which also highlight the burden of fatigue among individuals with similar health challenges (Fagerström & Frisman, 2017; Hjorth et al., 2020). The impact of fatigue was evident in participants’ daily functioning: for example, one participant was on disability benefits, while another attempted to maintain a work commitment of 20%. These experiences are consistent with the findings of Hjorth et al. (2020), which emphasize that fatigue significantly influences both social and work-related activities. Participants often had to navigate the complexities of maintaining social connections and job responsibilities while managing their energy levels, underscoring the need for supportive measures that can help them balance their health needs with their desire for engagement in daily life. The pervasive nature of fatigue among participants illustrates a critical component of living with chronic health issues. Their adaptive strategies highlight the importance of self-awareness and planning in maintaining quality of life, suggesting a need for greater understanding and support for individuals facing similar challenges.

#### My Second Home- Building Partnership Roles

Several participants expressed a profound sense of being in their “second home” when visiting the nurse-led outpatient clinic, a sentiment notably reflected in their practice of addressing nurses by their first names. This familiarity and comfort stem from the continuity of care they receive, the nurses’ accessibility, and their unwavering commitment to go the extra mile for the patients’ convenience. The close relationship between patients and nurses can be understood within the framework of person-centred care, which emphasizes collaboration and shared understanding between these two partners (Santana et al., 2018). During outpatient consultations, it is essential for nurses to demonstrate attentiveness, respect, and genuine engagement. Moreover, involving patients in shared decision-making lays a solid foundation for their learning and coping processes (Alvsvåg, 2018; Eklund et al., 2019; Heggdal, 2013). Participants’ descriptions of the nurses’ qualities resonate with Martinsen (2006) philosophy, which views nursing care as relational, practical, and moral. According to this perspective, nurses should exhibit solidarity with their most vulnerable patients, fostering a morally responsible power dynamic between the nurse and the patient. For individuals living with liver cirrhosis and ascites, who may find themselves in particularly vulnerable situations, this responsibility becomes even more critical.

Exciting literature has corroborated that the nurse-patient relationship significantly influences patients’ trust in healthcare providers (Carbonneau et al., 2018; Day et al., 2015; Hjorth et al., 2020; Ramachandran et al., 2022). A strong, trusting relationship not only enhances the quality of care but also empowers patients to actively engage in their health management, ultimately leading to better health outcomes. The nurturing environment fostered by nurses in the outpatient clinic plays a pivotal role in the overall well-being of patients, underscoring the importance of relational care in the management of chronic conditions such as liver cirrhosis and ascites.

Awareness of patients’ experiences living with ascites is crucial for optimizing holistic, person-centred care, particularly given the rapid changes in disease trajectory that these patients often face (Hjorth et al., 2020). Consistent with findings from (Ramachandran et al., 2022) and Abdi et al. (2015), our participants highly valued the accessibility of nurse-led clinics and the tailored health information provided. This information was delivered in a way that considered individual health literacy levels, with one participant describing the experience as being “spoon-fed” information. Participants noted that nurses frequently employed illustrations to clarify complex disease-related information, acknowledging that such content can often be challenging to understand (Hjorth et al., 2020). The participants’ satisfaction with the follow-up in nurse-led clinics can be attributed to the ample time allocated for consultations and the use of clear, comprehensible language. This contrasts with the findings of Hjorth et al. (2023), which reported issues such as short consultation times and overly medicalized information that hindered patients from learning about their disease. Additionally, participants reported gaining new insights during each outpatient visit, highlighting the significant role of health-related education for patients with chronic diseases (Carbonneau et al., 2018; Day et al., 2015; Ramachandran et al., 2022). However, it is equally important for nurses to respect the preferences of patients who may not desire extensive amounts of health-related information, as individual information needs can vary widely (Day et al., 2015). Effective communication and educational approaches in nurse-led clinics can play a transformative role in enhancing the patient experience for those living with ascites. By recognizing the diverse needs and preferences of patients, healthcare providers can foster an environment that supports both learning and autonomy in managing their health.

Our participants expressed significant satisfaction with their involvement in decisions regarding the frequency of their follow-up care and the content of the consultations. This level of engagement allowed them to have a meaningful impact on the treatment they received, contrasting sharply with findings from Fagerström and Frisman (2017) and Hjorth et al. (2020), where many participants reported feeling excluded from their follow-up treatment processes. Moreover, participants conveyed a clear preference for face-to-face consultations over phone or video interactions, highlighting the value they place on personal engagement in their healthcare experience. This preference for in-person interactions may be influenced by the demographic characteristics of our sample, as the majority of participants were retired. Given this context, further investigation into the reasons behind this preference in different age groups and settings would be beneficial. The positive feedback from participants regarding their involvement in care decisions reinforces the importance of patient inclusion in treatment planning. Understanding and acknowledging their preferences can lead to a more personalized and effective healthcare experience.

### Strengths and Limitations of the Study

A notable strength of our study is the diverse participation of patients across various ages, genders, levels of ascites, etiologies, and durations since diagnosis, contributing to a broader representation of the patient group. While this diversity enriches the findings by capturing a broader range of experiences, it may also limit the applicability of the results to specific patient populations with a particular etiology. Participants were recruited from two different hospitals, further enhancing the study’s diversity. Additionally, the choice of interview locations made by the participants likely fostered a sense of safety and comfort, which is crucial for candid discussions. Observations made by the interviewer indicated that participants exhibited relaxed body language during face-to-face interviews, whereas the phone interview appeared less relaxed. This difference may be attributable to the lack of direct interpersonal connection, prompting reflection on the significance of such dynamics within interview settings. It’s important to note that our sample included more women than men, and a more balanced gender representation may have yielded different perspectives. This observation highlights the need for further research to explore how gender dynamics influence patient experiences and perceptions in similar contexts. We aimed to explore patients’ experiences living with cirrhosis-related ascites; however, it is essential to acknowledge that some of the symptoms reported by participants may also be associated with liver cirrhosis more generally. This overlap may complicate our understanding of the specific impacts of ascites on patients’ quality of life, as the symptoms attributed to liver cirrhosis can obscure the unique challenges posed by ascitic conditions. By employing reflexive thematic analysis and ensuring a diverse participant pool, this study provides valuable insights into patient experiences while also raising important questions about the nuances of data collection methods and representation.

### Implications for Policy and Practice

Nurse-led clinics that emphasize continuity of care and operate within established treatment algorithms represent a promising approach to enhancing the quality of care and improving patient satisfaction for individuals living with liver cirrhosis and ascites. Previous research has demonstrated that outpatient education plays a crucial role in enhancing patients’ knowledge, self-care skills, and treatment adherence, which in turn lead to improved patient outcomes and reduced rates of hospital admissions (McAfee, 2012; O’Connell et al., 2023). Furthermore, increased accessibility to care may alleviate the anxiety and distress experienced by patients grappling with serious illnesses characterized by unpredictable trajectories. In Norway, however, the implementation of nurse-led clinics remains limited. Therefore, additional efforts are necessary, as task shifting could significantly enhance the care process. Initial steps have been taken to empower nurses to perform paracentesis drainage, reflecting emerging practices from the UK that suggest such task shifting can lead to reduced hospital admissions and improved cost-effectiveness (Fabrellas i Padrès et al., 2023). To optimize the efficacy of nurse-led clinics, it is advisable that they are staffed by clinical nurse specialists or advanced practice nurses, as their advanced educational background has a direct impact on professional nursing practice (Fabrellas i Padrès et al., 2023). Moreover, within outpatient settings, it is essential for nurses to collaborate within an interprofessional team that includes hepatologists, nutritionists, health psychologists, physical therapists, and social workers. This collaborative framework is critical for enhancing the holistic quality of care for patients with liver cirrhosis-related ascites.

## Conclusion and Recommendations for Further Research

The participants reported that their experiences with ascites and liver cirrhosis significantly impacted both their physical and psychological well-being. In coping with the challenges of living with ascites, participants actively sought to maintain their dignity. As they adapted to treatment recommendations, they developed embodied knowledge and employed strategies to manage their diminished energy levels. Additionally, participants expressed a high degree of satisfaction with the nurse-led follow-up care, characterizing their attendance at these consultations as akin to returning to a “second home.” This sentiment was grounded in the continuity of care provided by nurses, their availability, commitment, patient education skills, and the involvement of patients in their care plans. Further research is warranted to explore patients’ satisfaction with outpatient follow-up care in greater depth. Such studies should investigate the different modalities of consultations and strive to achieve a more balanced gender representation in qualitative research.

## Appendix 1

### Interview Guide

**Qualitative Study—**Patients’ experiences living with liver cirrhosis induced ascites, and the follow-up care they receive in nurse-led outpatient clinics.

**Table:**

**Opening Question:** Could you tell me a little about yourself? Your age and how long you have lived with this disease? Have you been admitted for ascitic drainage?

**Table:**

**Main Question 1** • Can you tell me a bit about how you experience living with ascites? ○ **Follow-up Question 1:** How do you cope in your daily life? ○ **Follow-up Question 2:** What can you no longer manage? ○ **Follow-up Question 3:** What do you avoid doing that you used to do before?
**Main Question 2** • What do you think is the most challenging aspect of living with the disease? ○ You mentioned _____. Could you elaborate on this? ○ I would like to hear more about __________. ○ You mentioned __________. Can you tell me how that felt? (Family, social life, relationships, community, work, children, living conditions?)
**Main Question 3** • Can you tell me a bit about how you feel you are treated by the nurses when you come to the hospital’s outpatient clinic? ○ **Follow-up Question 1:** How do you feel about the initial greeting? ○ **Follow-up Question 2:** How do you experience the follow-up consultation? ○ **Follow-up Question 3:** What do you think about the duration of the consultation? (too short/too long?) ○ **Follow-up Question 4:** How often are your consultations scheduled?

**Table:**

**Main Question 4** • What would you wish to be the focus during the follow-up care? ○ **Follow-up Question 1:** Do you feel there is something that is not addressed?
**Main Question 5** • How do you find adhering to the recommendations you receive from the healthcare system? ○ **Follow-up Question 1:** Fluid restrictions? ○ **Follow-up Question 2:** Medication adjustments? ○ **Follow-up Question 3:** Low-sodium diet?
**Main Question 6** • How do you find the ascitic drainage? ○ **Follow-up Question 1:** Painful? Do you prefer being admitted or doing the drainage in the outpatient clinic?
**Main Question 7** • Can you describe how you would like to be followed up by nurses based on your experiences so far? ○ **Follow-up Question 1:** Face-to-face and why? ○ **Follow-up Question 2:** Digital follow-up, and if so, how? ○ **Follow-up Question 3:** Is there any other way you think would suit you? Telephone? Other alternatives for follow-up?

**Closing**

**Table:**

Is there anything you think we have missed discussing today? Is there anything additionally you wish to mention?
